# Retinal Adaptation to Changing Glycemic Levels in a Rat Model of Type 2 Diabetes

**DOI:** 10.1371/journal.pone.0055456

**Published:** 2013-02-08

**Authors:** Leif E. Johnson, Michael Larsen, Maria-Thereza Perez

**Affiliations:** 1 Department of Ophthalmology, Glostrup Hospital, Glostrup, Denmark; 2 University of Copenhagen, Faculty of Health and Medical Sciences, Copenhagen, Denmark; 3 Department of Clinical Sciences, Division of Ophthalmology, Lund University, Lund, Sweden; Univeristy of Melbourne, Australia

## Abstract

**Purpose:**

Glucose concentrations are elevated in retinal cells in undiagnosed and in undertreated diabetes. Studies of diabetic patients suggest that retinal function adapts, to some extent, to this increased supply of glucose. The aim of the present study was to examine such adaptation in a model of type 2 diabetes and assess how the retina responds to the subsequent institution of glycemic control.

**Methods:**

Electroretinography (ERG) was conducted on untreated Zucker diabetic fatty (ZDF) rats and congenic controls from 8–22 weeks of age and on ZDFs treated with daily insulin from 16–22 weeks of age. Retinal sections from various ages were prepared and compared histologically and by immunocytochemistry.

**Principal Findings/Conclusions:**

Acute hyperglycemia did not have an effect on control rats while chronic hyperglycemia in the ZDF was associated with scotopic ERG amplitudes which were up to 20% higher than those of age-matched controls. This change followed the onset of hyperglycemia with a delay of over one month, supporting that habituation to hyperglycemia is a slow process. When glycemia was lowered, an immediate decrease in ZDF photoreceptoral activity was induced as seen by a reduction in a-wave amplitudes and maximum slopes of about 30%. A direct effect of insulin on the ERG was unlikely since the expression of phosphorylated Akt kinase was not affected by treatment. The electrophysiological differences between untreated ZDFs and controls preceded an activation of Müller cells in the ZDFs (up-regulation of glial fibrillary acidic protein), which was attenuated by insulin treatment. There were otherwise no signs of cell death or morphological alterations in any of the experimental groups. These data show that under chronic hyperglycemia, the ZDF retina became abnormally sensitive to variations in substrate supply. In diabetes, a similar inability to cope with intensive glucose lowering could render the retina susceptible to damage.

## Introduction

Diabetes affects today approximately 347 million people worldwide, 90% of whom have the type 2 form (http://www.who.int/mediacentre/factsheets/fs312/en/). These figures are conservative since a significant number of people with type 2 diabetes are typically not diagnosed until several years after the onset of the disease. A major complication affecting a number of patients is diabetic retinopathy, which is clinically characterized by retinal vascular abnormalities such as microaneurisms, hemorrhages and neovascularization, eventually leading to visual loss (see reviews [1], [2]). There is, however, a delay of years or decades between the onset of diabetes and the development of microangiopathy. It is now believed that diabetic retinopathy is not, at least initially, a primary vascular disorder but that protracted damage to neuronal and glial components of the retina could be involved. This notion is supported by the demonstration of early subclinical anomalies, such as abnormal oscillatory potentials of the electroretinogram [3], which may reflect alterations that eventually contribute to microvascular retinopathy [1], [2].

In the retina, glucose uptake is not dependent on insulin and therefore intracellular glucose levels rise and fall with systemic glycemia [4], [5]. In diabetes, this is a confounding factor when trying to link abnormal retinal function to retinal disease. Specifically, functional abnormalities such as impaired dark adaptation can be reversed simply by raising blood glucose from normoglycemia to the patient’s habitual glycemic level [6], [7]. These anomalies may reflect mere adaptations to abnormal conditions rather than irreparable damage to the retina. Evaluations of retinal performance should therefore take extant and historic glycemia into account. In patients with diabetes, we have recently demonstrated protracted adaptation to normalized glycemia with a delay of 4 to 12 months [8]. We postulated that this may be a critical period during which the retina is more susceptible to developing microvascular damage, which in some patients manifests as an early worsening of diabetic retinopathy after institution of improved metabolic control [9].

To better characterize the dynamics of retinal adaptation in diabetes, one would need to examine the retina during defined periods of extended hyperglycemia and subsequently normalized glycemia. In the present study, we examined by electroretinography (ERG) the retinal function of the Zucker diabetic fatty (ZDF) rat. The ZDF rat is a model of type 2 diabetes that lives for months without severe weight loss and some of the other complications often seen in other diabetes models and therefore can be used to facilitate such studies. ZDF rats carry a leptin receptor defect (ZDF-Lepr*^fa^*) [10]. They begin to develop hyperglycemia at 5–7 weeks of age and insulin resistance at 7–10 weeks [11]. We found in these rats that the responses of photoreceptors to light stimuli were unexpectedly higher when compared to controls while the inner retina developed functional impairment. Lowering glycemia with insulin halted the progression of some inner retinal abnormalities. However, it also reduced the photoreceptoral responses, revealing an inability to quickly re-adapt metabolism, which correlates with some aspects of clinical findings.

## Methods

### Ethics Statement

Experiments were approved by the Supervisory Authority on Animal Testing of Denmark (Dyreforsøgstilsynet; permit 2007_561-1401) and the Ethical Committee on Animal Experiments in Malmö/Lund, Sweden (Malmö/Lunds Djurförsöksetiska Nämnd; permit M79-09) and animals were treated according to the recommendations of the Association for Research in Vision and Ophthalmology. All electrophysiological studies were performed under ketamine/xylazine anesthesia, and all efforts were made to minimize suffering.

### Animals

Male ZDF and ZDF congenic control rats (Lean) of 7–13 weeks of age were purchased from Charles River Laboratories (Sulzfeld, Germany) and were kept until an age of 16 to 43 weeks. Animals were maintained under a 12-hour light (≤140 lux)/12 hour dark cycle and provided with food (Purina 5008 rat chow; International Product Supplies Ltd., London, England) and water *ad libitum*.

A subset of ZDF rats was given daily insulin (ZDF-i) from 16 weeks of age, whereas the others were kept untreated (ZDF). Blood sampled from the lateral tail vein was used to measure glucose concentrations using a handheld glucometer (OneTouch UltraEasy; LifeScan Inc., Milpitas, CA, USA) or a glucose oxidase colorimetric assay with commercially available reagents (Sigma-Aldrich Inc., St. Louis, MO, USA). In untreated ZDFs, fed blood glucose levels were highly variable, so blood glucose measurements after overnight fasting were used to regularly monitor these animals on days when no other procedures were performed on them. Insulin treated ZDFs could not be fasted, and therefore fed values were obtained. After the observational period, animals were killed by CO_2_ asphyxiation and decapitation.

### Insulin Treatment

Glycemia was controlled in a subset of ZDFs by subcutaneous injection of a long acting human insulin analogue (Lantus; Sanofi Aventis, Bridgewater, NJ, USA) starting at week 16. In order to characterize the acute effects of lowering glycemia, the first ERGs were run no later than 2 hours after insulin administration. In separate experiments, it was found that a dose of 200 IU/kg was necessary to reduce glycemia to 12 mmol/L or less at 2 hours and that higher doses did not effectively reduce it further. To avoid risking hypoglycemic shock, 200 IU/kg were then chosen as the first dose at week 16. Animals whose blood glucose had not reached 12 mmol/L or less were excluded from this time-point but were still given subsequent daily injections and were examined for long term insulin effects. On subsequent days, the daily insulin dose could be adjusted (100–225 IU/kg) to maintain (fed) blood glucose levels between 5 and 10 mmol/L.

The anaesthetic xylazine causes transient hyperglycemia [12]. A group of Lean rats (separate from those used for comparison to ZDF rats) were tested with ERG in two sessions: i) when they were only anaesthetized (Lean), and ii) when they were first given a subcutaneous injection of human insulin (2 IU/kg, Insulatard; Novo Nordisk, Bagsvaerd, Denmark) 30 minutes before administering anaesthesia (Lean-i). The recording sessions were made one week apart and the animals (n = 8) were 14 and 15 weeks at the time. Blood glucose was measured both prior to all injections and during the ERG.

### Electroretinography

Full field electroretinographs (ERGs) were performed periodically in all 3 groups (Lean, ZDF, ZDF-i). One group of ZDF rats was followed from 8 through 22 weeks of age. At 16 weeks, this group was divided and one of the subsets was given daily insulin (ZDF-i), whereas the other was kept untreated (ZDF). Additional animals were incorporated into all 3 groups and tested first at week 16 and followed until week 22. The number of animals in each experimental group tested at each time-point is given in Table 1.

**Table 1:** Number of animals examined by ERG for each time-point.

Age (weeks)	ZDF	Lean	ZDF-i
**8**	13	7	
**10**	14	6	
**12**	14	8	
**14**	14	7	
**16**	13	9	8
**19**	10	9	10
**22**	10	7	4

ZDF, Zucker Diabetic Fatty rats; Lean, congenic control rats; ZDF-i, insulin treated ZDF.

Rats were dark-adapted overnight and prepared for ERG recordings under dim red light. Following anesthesia with ketamine (85 mg/kg) and xylazine (4 mg/kg), animals were placed on a heated platform to maintain body temperature at 38°C. One drop each of tropicamide (1%), phenylephrine (5%) and oxybuprocaine (0.4%) were administered to each eye for dilation and topical anesthesia. Gold ring corneal electrodes were referenced to an inactive electrode placed in the mouth and a needle was inserted subcutaneously in the tail as a ground. ERGs were recorded using a Ganzfeld bowl equipped with LED and xenon lamps (model Q450 SCX; Roland Consult, Siegburg, Germany) and the VikingSelect analysis system (Nicolet Biomedical Instruments, Madison, WI, USA). Responses were filtered (0.2 Hz–1 KHz, no notch filter) and digitized at 2.5 KHz over a 400 msec interval. Scotopic ERGs were recorded for stimuli of −3.7, −3.0, −2.0, −1.0, 0.0, 0.5 and 1.0 log cd*s/m^2^. After 5 minutes of light adaptation to a 1.53 log cd/m^2^ white background light, photopic ERGs were recorded for stimuli of 0.0, 0.5, 1.0, 1.3 and 1.5 log cd*s/m^2^. The photopic b-waves were heavily dominated by oscillatory potentials and so the same oscillation was used for each intensity to determine b-wave peaks for every trace. For stimuli of −1.0 log cd*s/m^2^ and less, the interstimulus interval was 5 seconds and for stimuli above −1.0 log cd*s/m^2^, it was 17 seconds. Ten responses were averaged for the lower intensities and 5–10 for the higher intensities.

### Tissue Preparation and Histology

Retinal tissue was obtained from Lean as well as untreated and treated ZDF rats for analysis. Eyes were quickly enucleated following decapitation and immersed in a solution of 4% paraformaldehyde in Sørensen’s buffer overnight at 4°C. They were subsequently rinsed, cryo-protected in the same buffer containing increasing concentrations of sucrose, embedded in an albumin-gelatin medium, frozen and stored at −80°C. Sections were obtained on a cryostat (12 µm), collected on gelatin/chrome alum-coated glass slides, air-dried, and stored at −20°C until further processing. Tissue was taken at different ages (Lean: 16 (n = 4), 23 (n = 4), 31 (n = 2) weeks old; untreated ZDF (ZDF): 16 (n = 4), 23 (n = 3), 31 (n = 3) and 43 (n = 2) weeks old; treated ZDF (ZDF-i): 16 (n = 4) and 23 (n = 6) weeks old). Treated animals were killed no later than 2 hours after insulin administration.

For the morphological analysis, sections were stained with hematoxylin and eosin and were visualized using bright field illumination (Axiophot, Carl Zeiss Meditec, Inc., Oberkochen, Germany). Cell rows of the outer and inner nuclear layers were counted in 3 fields of the mid-peripheral region of sections crossing the center of the eye (3 sections/animal) and averaged for each animal at 23 weeks of age. Images were taken with a digital camera and accompanying software (Axiovision 4.2, Carl Zeiss Meditec, Inc., Oberkochen, Germany). Unilateral retinal atrophy was noted in one eye of two ZDF rats, which resulted in substantially lower or flat ERGs. The atrophy was confirmed by morphological analysis and was characterized by thinning of the retina. These eyes were excluded from the electrophysiological and morphological analyses.

### TUNEL Assay

Dying cells were detected in retinal sections with a terminal deoxynucleotidyl transferase dUTP nick end- labeling (TUNEL) assay, employing the *In Situ* Cell Death Detection Kit, TMR red (Roche Diagnostics, Mannheim, Germany), as previously described [13]. Briefly, the enzyme solution was diluted 1∶9 and the labeling solution 1∶4 in PBS. The two components were mixed 1∶4.44 immediately before application to the sections for 45 minutes at 37°C, after which the reaction was stopped by several washes with cold PBS. Sections were mounted with anti-fading medium VECTASHIELD® (Vector Laboratories, Burlingame, CA, USA) and examined with a fluorescence microscope (Axiophot, Carl Zeiss Meditec, Inc., Oberkochen, Germany). Images were taken with a digital camera and accompanying software (as above). The entire span of 3 sections was examined for each animal taken for histology (see above) at 16 and 23 weeks of age. The number of TUNEL positive cells for each retinal layer was averaged for all 3 groups.

### Immunocytochemistry

Retinal sections (from each animal taken for histology, see above) were blocked and permeabilized by pre-incubation with PBS (10 mM, pH 7.2) containing 0.25% Triton X-100 (PBS-T), 1% bovine serum albumin (PBS-TA) and 5% normal serum, followed by overnight incubation at 4°C with PBS-TA containing 1% normal serum and one of the following: a rabbit polyclonal antibody against glial fibrillary acidic protein (GFAP; 1∶1500; DAKO A/S, Glostrup, Denmark), a rabbit monoclonal antibody against phospho-Akt (Ser473) (pAkt; 1∶30; Cell Signaling Technology, Danvers, MA, USA). Sections were then washed and incubated with a secondary antibody, DyLight-488 donkey anti-rabbit (Jackson ImmunoResearch Laboratories Inc, West Grove, PA, USA) or Alexa Fluor 488 goat anti-rabbit (Molecular Probes, Eugene, OR, USA) at 1∶200 for 60 min at room temperature. The sections were subsequently rinsed, mounted with VECTASHIELD containing the nucleic acid stain 4′,6-diamidino-2-phenylindole (DAPI) (Vector Laboratories, Burlingame, CA, USA). Negative controls were obtained by omitting the primary antibody. The sections were examined with a fluorescence microscope (as above) and images acquired with a digital camera and accompanying software (as above) using the same illumination and acquisition settings for all sections processed with the same antibody.

### Data Analysis

Using the Nicolet VikingSelect software (version 11.1), the trough of the a-wave and peak of the b-wave were marked to determine the implicit times and amplitudes of responses. This information and the ERG trace raw data were then exported into Microsoft Office Excel (2003) for further analysis.

The b/a ratio for scotopic responses was calculated by dividing b-wave amplitudes by a-wave amplitudes for each animal at −1.0, 0.0, 0.5 and 1.0 log cd*s/m^2^. The slope of the falling phase of the scotopic a-wave was determined at each time point by calculating change in amplitudes recorded one sampling instance prior to and after that time point, then dividing it by 2 to get the change in amplitude at that time point. The most negative value returned for this segment was then entered as the maximal a-wave slope for that ERG trace.

Oscillatory potentials (OPs) were extracted from scotopic responses to a stimulus of 0.0 log cd*s/m^2^ with a band-pass filter of 75–300 Hz by using an add-in function for Microsoft Office Excel (www.web-reg.de/bp_addin.html#).

For comparisons of all parameters of Lean, ZDF, and ZDF insulin-treated rats of 8–22 weeks of age, two-way analysis of variance (ANOVA) was applied to determine statistical significances of the interaction between experimental group and age using the MIXED procedure in SAS (version 9.1; SAS Institute, Cary, NC, USA). Significant differences between experimental groups at each age were determined by the Least Significant Difference test (LSD). For cell row number and TUNEL positive cell counts, one-way ANOVA was applied using the same program. For comparisons of insulin treated and untreated Lean rats, paired, two-tailed t-tests (Microsoft Office Excel) were performed. Data are presented in Tables as ± SD and differences were considered statistically significant when p<0.05.

## Results

### Glycemia and Body Weight

**Figure 1:**
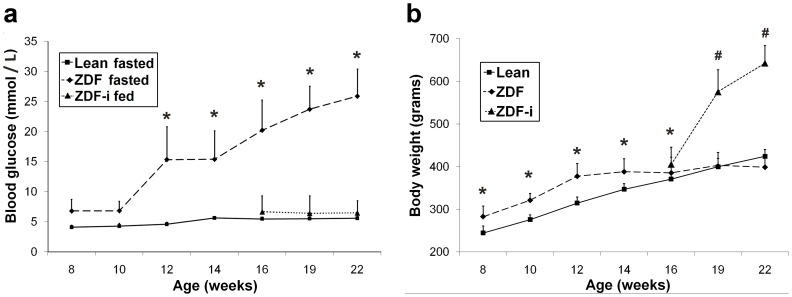
Average blood glucose and body weight as a function of age. ZDF rats were hyperglycemic in the fed state at 8 and 10 weeks of age, but measurements were highly variable. Blood glucose (**a**) in untreated ZDF (*dashed line*) and Lean (*solid line*) rats was measured in the fasted state. Treated ZDFs (ZDF-i) could not be fasted, and therefore fed values were obtained (*dotted line*). Body weights (**b**) of ZDF (*dashed line*), Lean (*solid line*) and ZDF-i rats (*dotted line*) are shown. Data presented are group mean +SD of 4–14 animals and significant differences (2 way ANOVA LSD; p<0.05) between ZDFs and Leans are denoted by *and between ZDF-i and the other experimental groups by **^#^**ZDF-i, insulin-treated ZDFs.

Average fasted glucose levels of Lean rats were normal and remained constant throughout the study. ZDF rats were hyperglycemic (blood glucose >11 mmol/L) in the fed state at weeks 8 and 10 (not shown), but were still relatively normoglycemic when fasted (Fig. 1a). From 12 weeks of age, however, glucose levels were significantly higher than in Leans also in the fasted state (p<0.05; Fig. 1a). Insulin treatment lowered glycemia and ZDF-i rats were kept at 5–10 mmol/L (fed state) for the duration of the study. Untreated ZDFs initially weighed more than Leans (p<0.05) while insulin treated ZDFs weighed significantly more than Leans and untreated ZDFs at weeks 19 and 22 (p<0.05; Fig. 1b).

### Electroretinography

A longitudinal study was performed on ZDF and Lean rats to characterize the progression of electrophysiological changes in the diabetic retina. A comprehensive list of data collected is provided in Tables S1, S2, S3, S4, S5 and averaged ERG and OP traces for experimental groups at representative ages and intensities are overlapped and shown in Figure S1.

**Figure 2:**
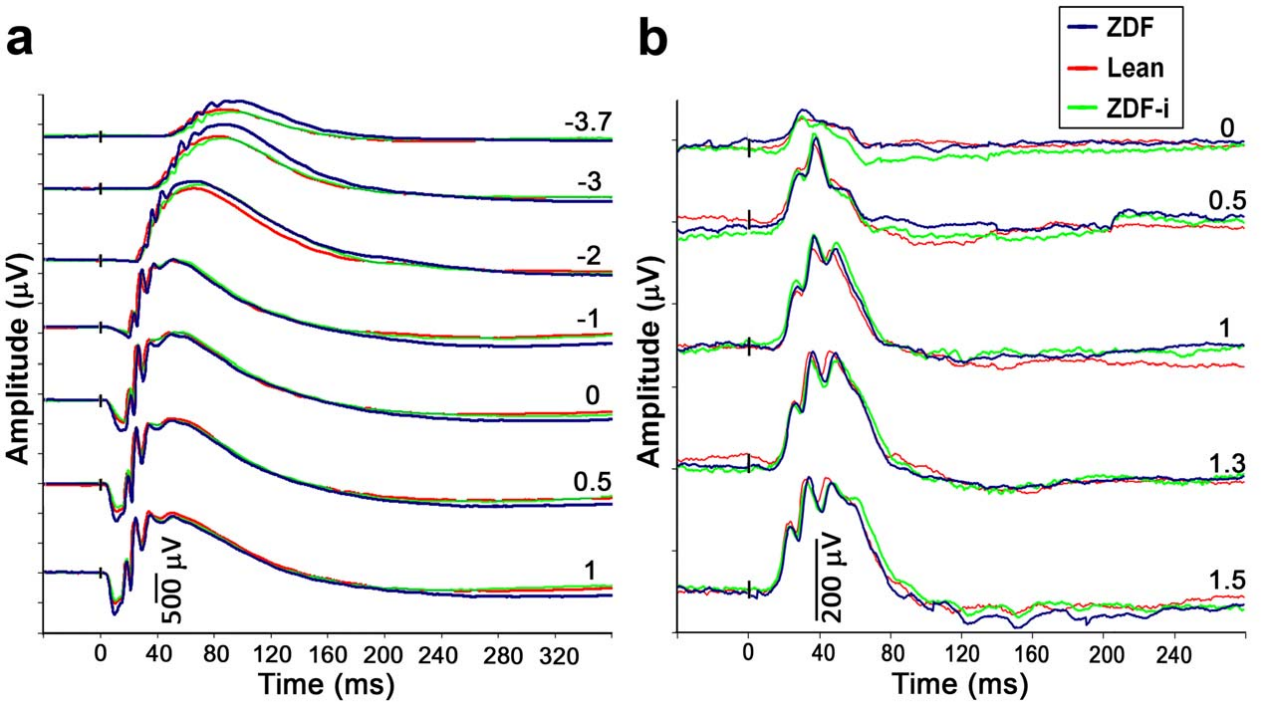
Electroretinograms (ERGs) of all experimental groups. A series of ERG traces were elicited by flashes of increasing intensity (denoted on right as value of log cd*s/m^2^ for each trace) under scotopic (**a**) and photopic (**b**) conditions. The traces shown are from an animal representative of each experimental group at 16 weeks of age. ZDF-i, insulin-treated ZDFs.

ERGs were recorded within 15–30 minutes after sedation. Lean rats had fed blood glucose levels of 5.9±0.7 mmol/L prior to anaesthesia and xylazine caused these levels to rise to 16.9±1.2 mmol/L for the duration of recordings. The glycemic levels of anesthetized ZDF rats were, however, unaffected as were those of insulin treated ZDFs which remained at 5–12 mmol/L.ERG measurements were made under dark-adapted (scotopic) and light-adapted (photopic) conditions periodically between 8 (approximately 2 weeks after the onset of hyperglycemia in ZDF rats) and 22 weeks of age, after which untreated diabetic animals start to develop visible cataracts. At week 16, fed blood glucose levels fell to 10.4±1.6 mmol/L in ZDF-i rats within 2 hours of insulin administration, at which point ERGs were recorded. ERG traces representative for the three groups under scotopic and photopic conditions are shown in Figures 2a and 2b, respectively. Treated ZDFs were subsequently given a daily dose of insulin and ERGs on these animals were conducted 2 hours after administration at weeks 19 and 22.

#### Transient hyperglycemia does not affect ERG responses in normal rats

As mentioned above, anesthesia induced an increase in glucose levels in Lean rats, which lasted for the duration of the ERG recordings. To assess whether this acute hyperglycemia had an effect on ERG responses, a control experiment was conducted with a separate group of Lean rats, which in one session received insulin 30 minutes prior to anaesthesia (Lean-i). Before any injection, these rats had an average blood glucose of 6.3±0.45 mmol/L, while during the ERGs (under anaesthesia) their blood glucose rose to 16.5±2.0 mmol/L. On a second session, insulin administration prevented the rise in glycemia (average 5.7±1.2 mmol/L) but did not produce any noteworthy changes in ERG responses (Table 2).

**Table 2:** Effect of insulin on control Lean rat ERG.

Scotopic responses						
Intensity:	−3.7		−3.0		−2.0	
Group:	Lean	Lean-i	Lean	Lean-i	Lean	Lean-i
b-wave amplitude	501±98	457±129	945±123	890±132	1198±120	1171±65
b-wave IT	87.9±3.7	89.2±3.5	84.6±3.5	86.4±3.0	64.6±1.8	64.7±1.9
	**−1.0**		**0.0**		**0.5**	
	**Lean**	**Lean-i**	**Lean**	**Lean-i**	**Lean**	**Lean-i**
a-wave amplitude	141±8	144±21	390±21	390±41	462±31	455±42
b-wave amplitude	1178±118	1175±59	1407±114	1429±68	1416±123	1429±70
a-wave IT	18.8±0.5	18.9±0.1	16.4±0.4	16.5±0.3	10.7±0.3	10.7±0.2
b-wave IT	51.2±0.5	**52.5±1.3** *	50.5±0.8	52.5±2.6	50.1±1.0	**51.2±1.5** *
	**1.0**					
	**Lean**	**Lean-i**				
a-wave amplitude	494±30	499±42				
b-wave amplitude	1335±103	1361±81				
a-wave IT	9.7±0.2	9.8±0.3				
b-wave IT	51.5±1.7	52.1±1.8				
**Photopic responses**						
**Intensity:**	**0.0**		**0.5**		**1.0**	
**Group:**	**Lean**	**Lean-i**	**Lean**	**Lean-i**	**Lean**	**Lean-i**
b-wave amplitude	73±14	84±15	193±21	182±17	233±19	**211±21** *
b-wave IT	29.7±0.7	29.8±0.7	37.1±0.8	37.4±0.6	36.5±0.9	36.7±0.7
	**1.3**		**1.5**			
	**Lean**	**Lean-i**	**Lean**	**Lean-i**		
b-wave amplitude	248±16	**226±27** *	249±21	239±22		
b-wave IT	48.5±1.4	49.1±1.5	46.6±1.8	47.5±1.4		

Units for intensity denoted as log cd*s/m^2^; Data presented are group mean ± SD; Amplitude denoted in µV; Implicit times (IT) denoted in ms; Age denoted in weeks.

Lean, congenic control rats; Lean-i, insulin treated Lean rats;

* p<0.05; n = 8.

#### Scotopic photoreceptoral responses are higher in diabetic rats and reduced by insulin

**Figure 3:**
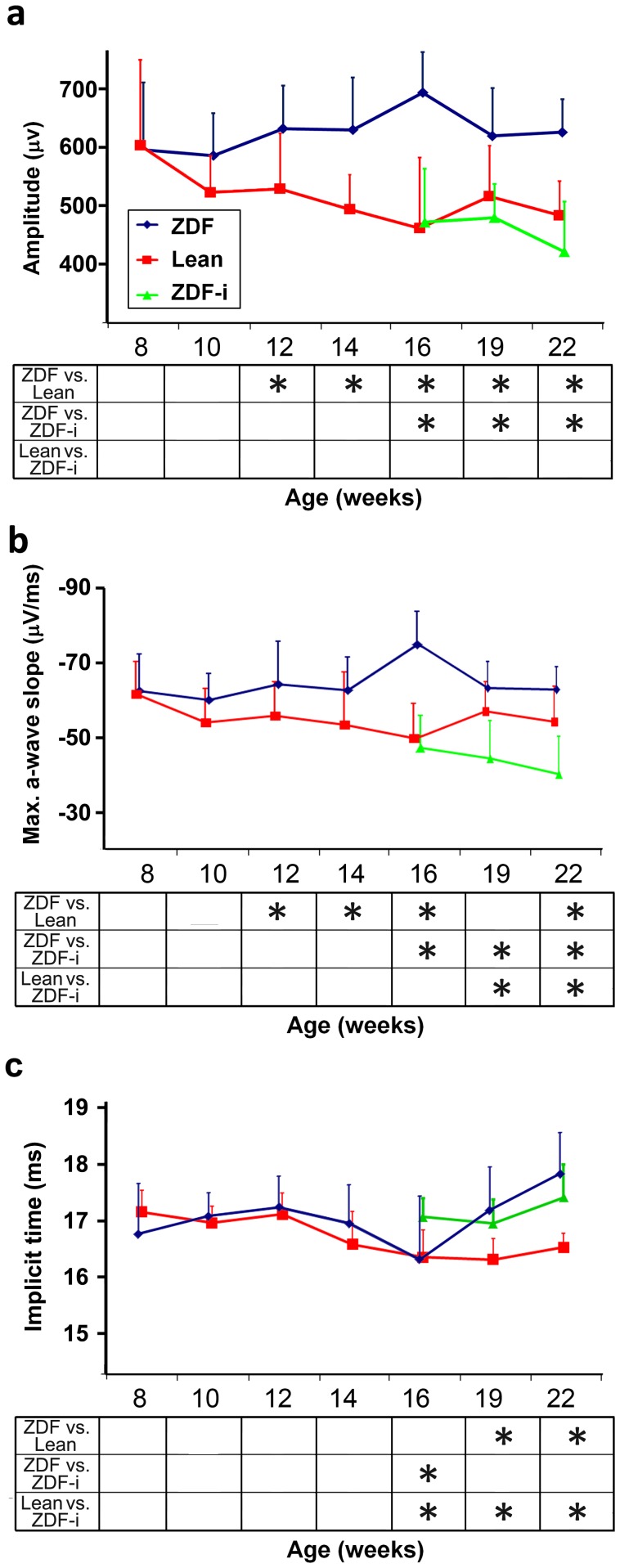
Scotopic a-wave parameters. Photoreceptor responses are characterized by a-wave amplitude (**a**), maximum slope (**b**) and implicit times (**c**). Responses shown in (**a**) and (**b**) are to 1 log cd*s/m^2^ and to 0 log cd*s/m^2^ in (**c**). Data presented are group mean +SD (see Table 1 for number of animals in each group at various ages) and significant differences (2 way ANOVA LSD; p<0.05) between individual pairs of experimental groups at each age tested are indicated (*) in tables below the graphs. ZDF-i, insulin-treated ZDFs.

Outer retinal responses were characterized by the amplitude, implicit time and maximum slope of a-waves (complete data in Table S1). These were all similar in Leans and ZDFs at week 8, after which the a-wave amplitudes in Leans showed a tendency to diminish with increasing age (not statistically significant, p>0.05). In contrast, ZDF a-wave amplitudes remained elevated and for weeks 12 through 22 were approximately 20% larger than those of Leans (p<0.05) (Figs. 2c, 2e; 3a). This change was paralleled by larger, though in general not significant, maximum slopes in ZDFs (Fig. 3b). Implicit times of a-waves at all but the lowest intensity showed no differences until weeks 19 and 22 when a delay was detected in ZDFs (p<0.05) compared to Leans (Fig. 3c).

At week 16, a subgroup of ZDFs received insulin for the first time, while the others remained untreated. Insulin administration had an immediate effect on outer retinal responses (complete data in Table S1). The a-wave amplitudes of treated ZDFs were about 30% lower (p<0.05) than untreated ZDFs and similar to those of Leans for all stimuli intensities (Fig. 3a). The maximum a-wave slopes for stimuli of 0.0 log cd*s/m^2^ or higher were reduced when compared to untreated ZDFs (p<0.05) and again similar to Leans (Fig. 3b). The a-wave implicit times of treated and untreated ZDF rats were not consistently different (Fig. 3c).

In the treatment group, ZDFs continued to receive insulin injections daily for the following 6 weeks. Insulin administration was associated with a sustained reduction of the outer retinal responses (complete data in Table S1). Treated ZDF amplitudes were about 20–35% lower than untreated ZDFs at weeks 19 and 22 (p<0.05; Fig. 3a). Although they were also markedly lower than those of Leans, the differences between these groups were not statistically significant (Fig. 3a). The maximum slopes of treated ZDFs were also reduced when compared to both untreated ZDFs and Leans for all but the lowest stimulus intensity (p<0.05; Fig. 3b). Implicit times were not affected when compared to untreated ZDFs, but tended to be slower than Leans (Fig. 3c).

#### Scotopic b-waves are not proportional to a-waves after prolonged hyperglycemia

**Figure 4:**
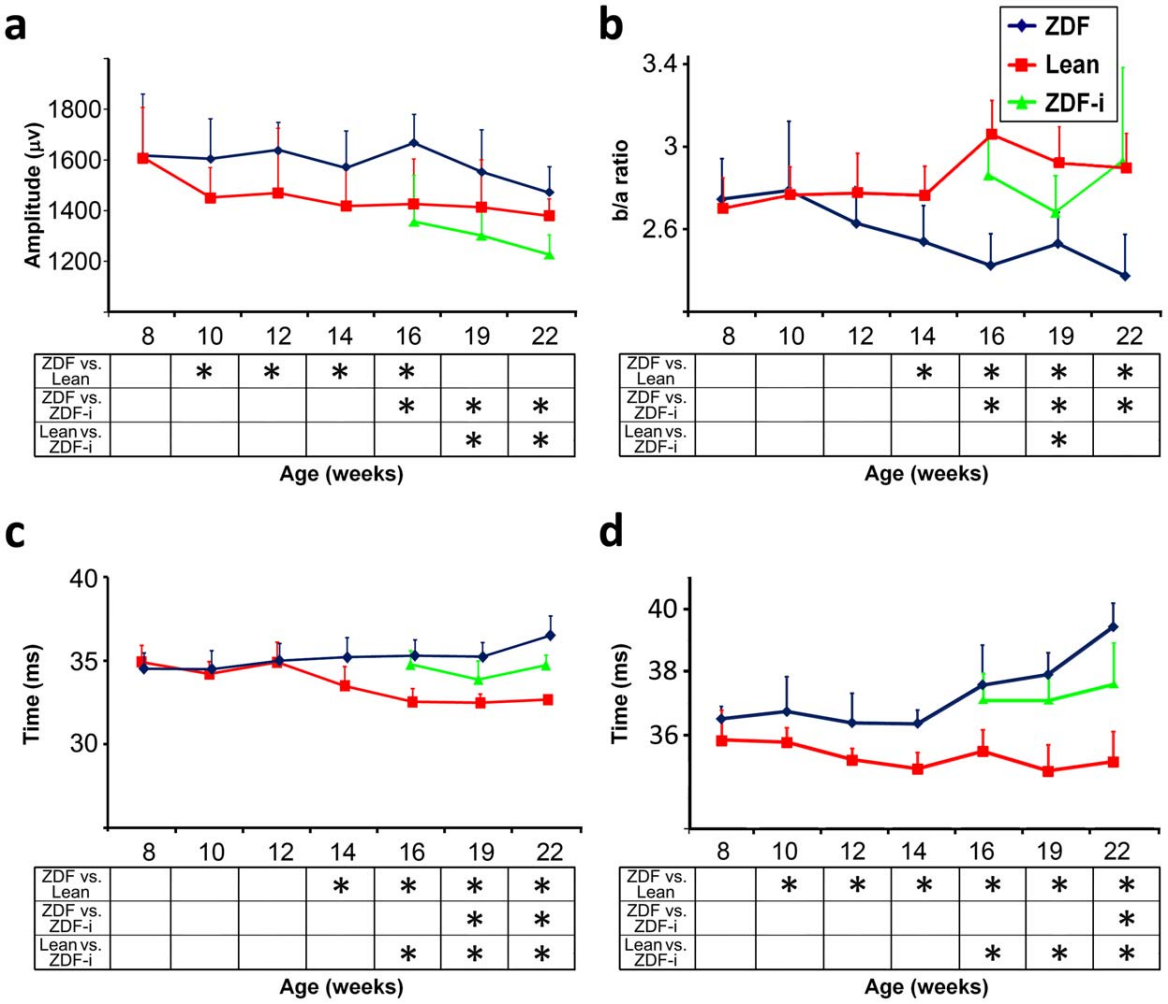
Inner retinal parameters. Scotopic b-wave amplitudes (**a**) and b/a ratio (**b**) for 1 log cd*s/m^2^ are shown. Implicit times for oscillatory potential wavelet 3 (c) and the photopic b-wave (d) are also shown (0 and 1 log cd*s/m^2^, respectively). Data presented are group mean +SD (see Table 1 for number of animals in each group at various ages) and significant differences (2 way ANOVA LSD; p<0.05) between individual pairs of experimental groups at each age tested are indicated (*) in tables below the graphs. ZDF-i, insulin-treated ZDFs.

Scotopic b-wave amplitudes (complete data in Table S2A) of ZDFs were similar to Leans at week 8 but were larger thereafter (Fig. 4a). At weeks 12, 14 and 16, ZDF amplitudes for stimuli of −2.0 to 1.0 log cd*s/m^2^ were approximately 10–20% higher (p<0.05) than those of Leans (Fig. 4a). At weeks 19 and 22, ZDF amplitudes were still generally larger than Leans but these differences were not statistically significant (Fig. 4a).

To quantify these responses as a function of outer retinal activity under scotopic conditions, a b-wave to a-wave amplitude ratio (b/a ratio) was calculated (Table S2B). The two groups were similar at weeks 8 and 10, but the b/a ratio decreased progressively thereafter in ZDFs and increased in Leans after week 14. At weeks 14 through 22, ZDFs had significantly lower b/a ratios (p<0.05) for intensities 0.0 to 1.0 log cd*s/m^2^ (Fig. 4b). Scotopic b-wave implicit times (complete data in Table S3) were similar for Lean and ZDF rats at weeks 8 and 10, but generally slower for ZDFs (p<0.05), although not always statistically significant for all stimulus intensities.

Treating ZDF rats with insulin caused 10–20% decreases (p<0.05) in b-wave amplitudes (complete data in Table S2A) both acutely and long term in response to stimuli of −2.0 log cd*s/m^2^ and higher (Fig. 4a). Though comparable at week 16 (Fig. 4a), treated ZDF responses became significantly lower than those of Leans over time as well (p<0.05) (Fig. 4a). The b/a ratios of treated ZDFs were, however, higher than those of untreated ZDFs (p<0.05) and in general similar to those of Leans (Fig. 4b).

#### Insulin does not reverse the hyperglycemia-induced delay of oscillatory potentials

Oscillatory potentials (OPs) were extracted by filtering scotopic responses to a stimulus of 0.0 log cd*s/m^2^, resulting in the isolation of four distinct wavelets (OP1-OP4). Already at week 10, OP implicit times of ZDF rats began to show a delay when compared to Leans. At week 14 and afterwards, all four wavelets were significantly slower in ZDFs (p<0.05) (Table 3; Fig. 4c). OP implicit times of treated ZDFs were in general shorter than those of untreated ZDFs (p<0.05) but remained slower than Leans at all time-points (p<0.05; Table 3). The OPs are a product of the activity of the whole retina and we therefore normalized the amplitudes by dividing them with the slope of the a-wave [14]. The interaction between experimental group and age was not significant for most wavelets and so no consistently significant differences were observed (Table S4).

**Table 3:** Scotopic oscillatory potential implicit times.

OP number:		1			2	
Group:	Lean	ZDF	ZDF-i	Lean	ZDF	ZDF-i
Age						
8	20.7±0.6	21.0±0.4		26.2±0.7	26.5±0.6	
10	20.3±0.2	20.9±0.6*		25.7±0.2	26.4±0.7*	
12	20.6±0.6	21.1±0.6*		26.2±0.7	26.8±0.6*	
14	19.7±0.6	21.2±0.6*		25.2±0.7	26.9±0.8*	
16	19.7±0.5	21.2±0.4*	20.5±0.5† ^#^	24.8±0.6	27.0±0.8*	26.0±0.4† ^#^
19	19.7±0.5	21.4±0.6*	20.3±0.7† ^#^	24.9±0.5	27.2±0.6*	25.6±0.7† ^#^
22	19.9±0.5	21.8±0.4*	21.3±0.5†	25.2±0.4	28.0±0.7*	26.8±0.7† ^#^
		3			4	
	Lean	ZDF	ZDF-i	Lean	ZDF	ZDF-i
8	34.9±1.0	34.5±0.9		48.2±1.7	47.7±1.5	
10	34.2±0.7	34.5±1.1		47.8±1.2	47.6±1.5	
12	34.9±1.2	35.0±1.0		48.6±1.4	48.5±1.6	
14	33.5±1.1	**35.2±1.2** *		46.9±1.6	**48.4±1.6** *	
16	32.5±0.8	**35.2±1.4** *	**34.6±0.5** †	45.6±1.1	**48.3±2.0** *	**48.3±0.8** †
19	32.5±0.5	**35.2±0.8** *	**33.3±1.0** † ^#^	45.0±0.5	**48.3±1.3** *	**46.9±1.6** †
22	32.7±0.2	**36.3±0.8** *	**34.5±0.6** † ^#^	45.7±0.6	**49.6±1.2** *	**47.1±0.6** #

Responses elicited by intensity of 0.0 log cd*s/m^2^; Data presented are group mean ± SD (see Table 1 for number of animals in each group at various ages); Implicit times (IT) denoted in ms; Age denoted in weeks.

OP, oscillatory potential; ZDF, Zucker Diabetic Fatty rats; Lean, congenic control rats; ZDF-i, insulin treated ZDF.

* p<0.05 between Lean and ZDF.

† p<0.05 between Lean and ZDF-i.

# p<0.05 between ZDF and ZDF-i.

#### Photopic b-waves are delayed in ZDF rats

Cone-dependent responses were assessed by measuring retinal function under photopic conditions (complete data in Table S5). The implicit times of photopic b-waves were longer in ZDFs at all time-points after week 10 (p<0.05; Fig. 4d). However, in contrast to scotopic ERGs, no consistent differences in photopic b-wave amplitudes were found between ZDF and Lean rats. Photopic b-wave implicit times were initially not affected by insulin, and were still longer in treated ZDFs than in Leans at weeks 16 and 19. At week 22, however, treated ZDFs had implicit times which were shorter than untreated ZDFs (p<0.05) but still longer than Leans (p<0.05; Fig. 4d). Decreases in some b-wave amplitudes were noted in insulin treated ZDFs.

### Retinal Morphology Appears Normal in ZDF Rats

**Figure 5:**
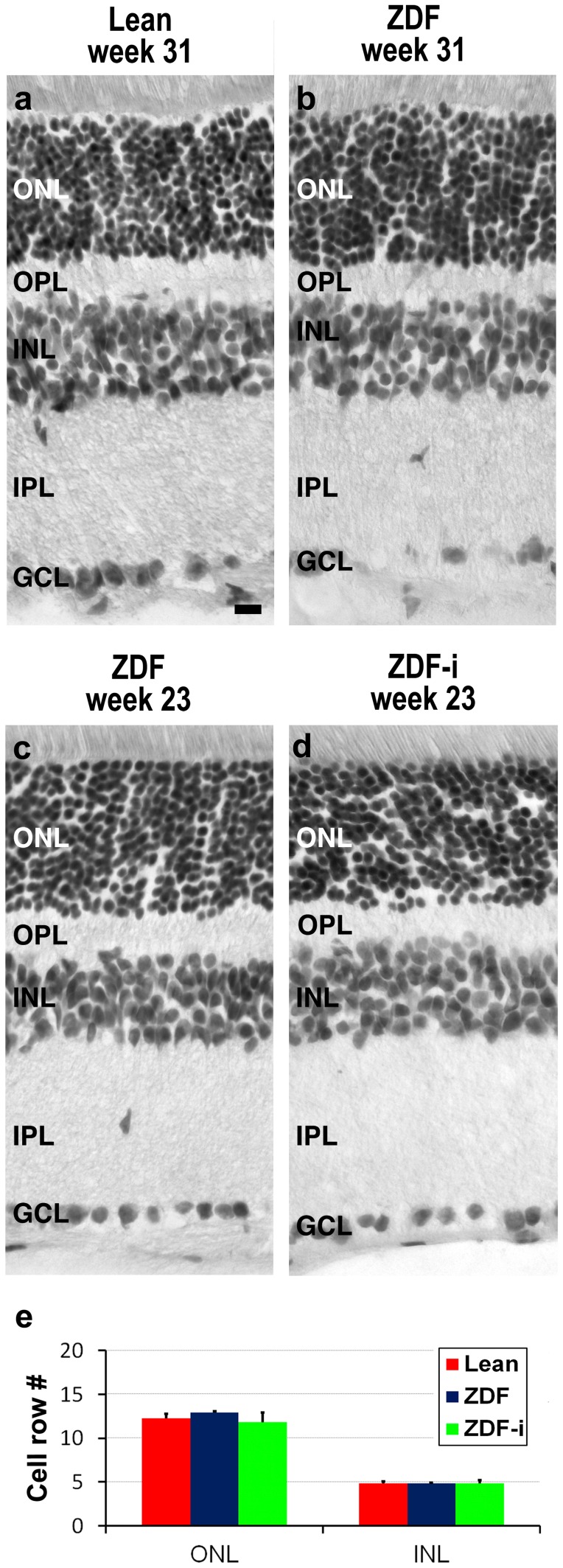
Retinal morphology. Hematoxylin and eosin stainings are shown for cryostat sections obtained from 31-week-old Lean (**a**) and ZDF (**b**) rats and from untreated (**c**) and insulin treated (**d**) ZDF rats at 23 weeks of age. The average number+SD of cell rows (**e**) in ONL and INL at 23 weeks of age were calculated for Lean (n = 4), untreated ZDF (n = 3) and insulin-treated ZDF (n = 6) rats, and no significant differences were detected (one way ANOVA; p>0.05). ZDF-i, insulin-treated ZDFs; ONL, outer nuclear layer; OPL, outer plexiform layer; INL, inner nuclear layer; IPL, inner plexiform layer; GCL, ganglion cell layer. Scale bar = 10 µm.

**Figure 6:**
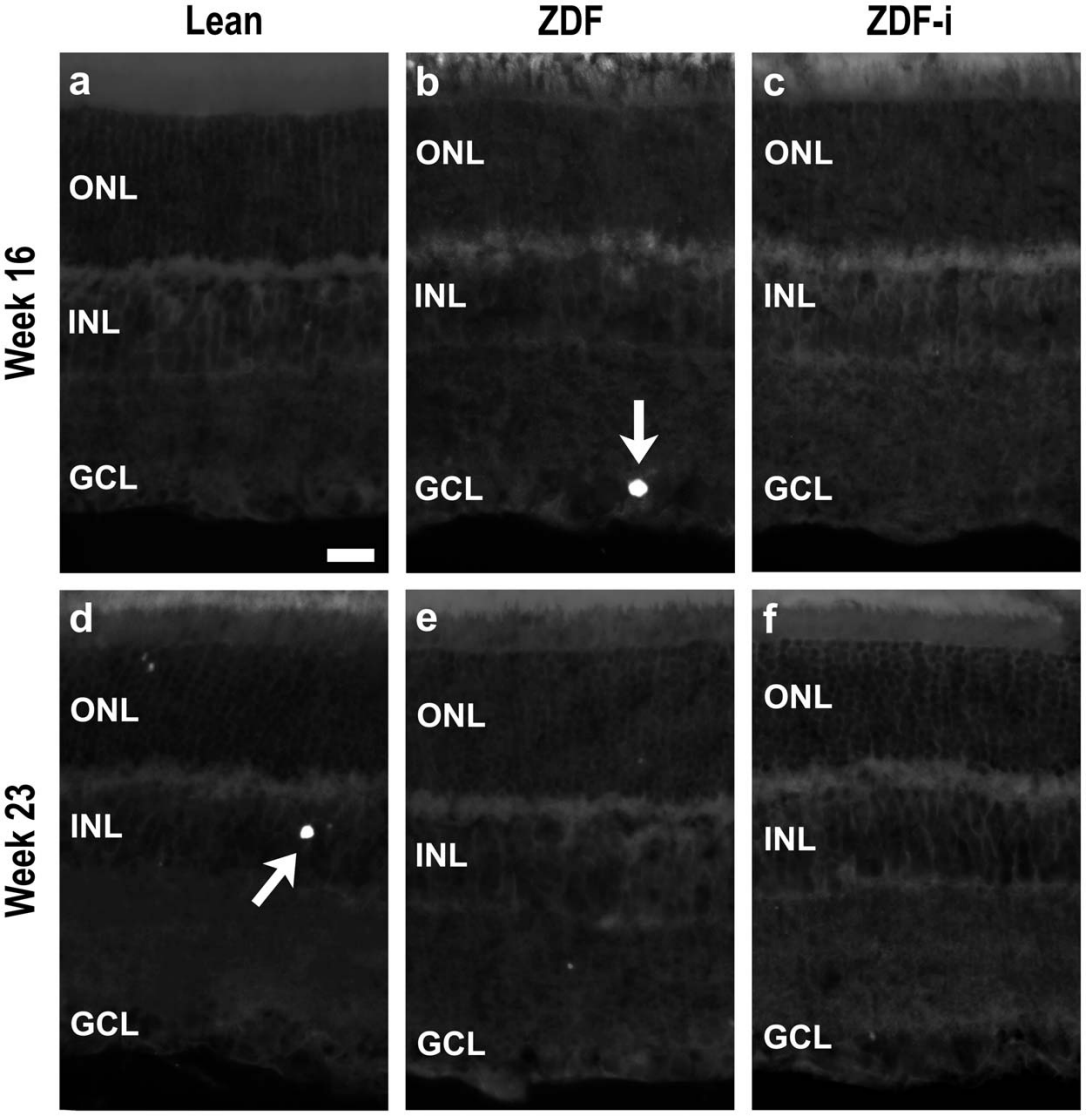
Cell death. TUNEL staining of cryostat sections obtained from Lean rats at 16 (**a**) and 23 (**d**) weeks of age, from ZDF rats at 16 (**b**), and 23 (**e**) weeks of age, and from insulin treated ZDF rats at 16 (**c**) and 23 weeks of age (**f**)**;** arrows point to positive cells found in ZDF (**b**) and Lean (**d**) rats. The average number of TUNEL positive cells +SD in each retinal layer were calculated (**g**) for Lean, untreated ZDF and insulin-treated ZDF rats at 16 (n = 4) and 23 weeks of age (n = 4, 3 and 6 respectively), and no significant differences were detected at either age (one way ANOVA; p>0.05). ZDF-i, insulin-treated ZDFs; ONL, outer nuclear layer; INL, inner nuclear layer; GCL, ganglion cell layer. Scale bar = 20 µm.

A comparison of retinal sections obtained from all animals at the available ages revealed no obvious morphological alterations such as abnormal changes in retinal thickness or a disruption of the layered structure of the retina (Figs. 5a–d). A quantification of the number of cell rows in the outer and inner nuclear layers of retinas in all groups at 23 weeks of age (Fig. 5e) also showed no significant differences. Cell death was evaluated using the TUNEL assay and revealed the presence of occasional positive cells in retinas of all groups (Figs. 6a–f), with no significant differences between any of them at 16 or 23 weeks of age (Fig. 6g).

### pAkt Distribution is Unaltered but GFAP Expression is Increased in Diabetic Retinas

**Figure 7:**
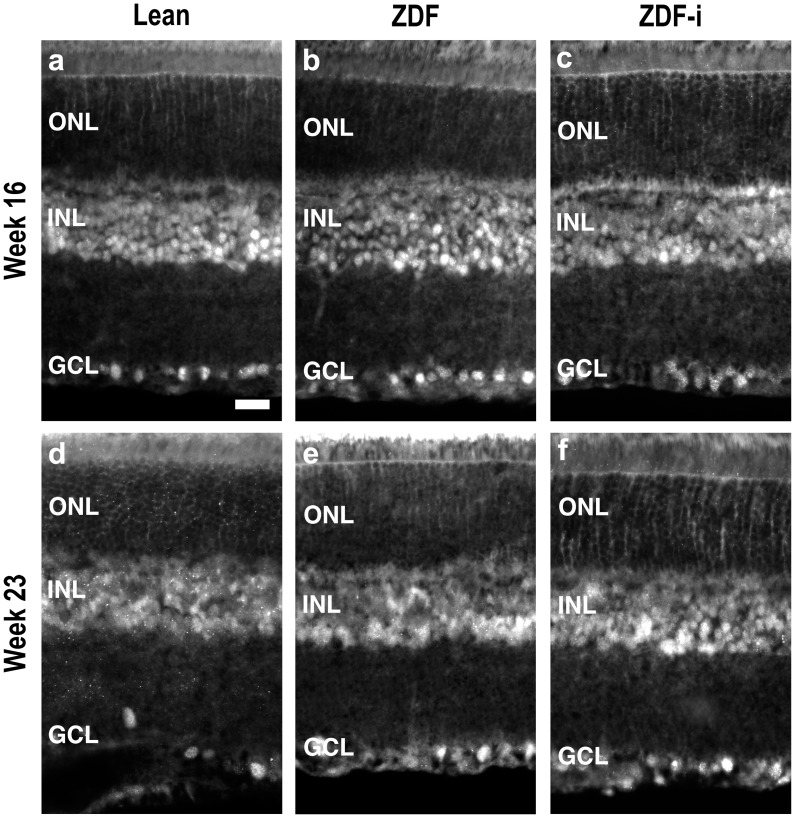
Phosphorylated Akt (pAkt) immunofluorescence. A phospho-specific antibody was used to detect activated Akt in the retina of Lean (**a**), ZDF (**b**) and insulin treated ZDF (**c**) rats at 16 weeks of age; and of Lean (**d**), ZDF (**e**) and insulin treated ZDF (**f**) rats at 23 weeks of age. ZDF-i, insulin-treated ZDFs; ONL, outer nuclear layer; INL, inner nuclear layer; GCL, ganglion cell layer. Scale bar = 20 µm.

Activation of insulin receptors present in the retina leads to an up regulation of pAkt [15]. The distribution of pAkt was compared in retinas from Lean rats (weeks 16, 23, 31) and from untreated (weeks 16, 23, 31, 43) and insulin-treated (weeks 16, 23) ZDFs. The protein was localized primarily in cells in the inner nuclear layer and in the ganglion cell layer and no consistent differences were observed between any of the experimental groups at the different time points (Figs. 7a–f).

**Figure 8:**
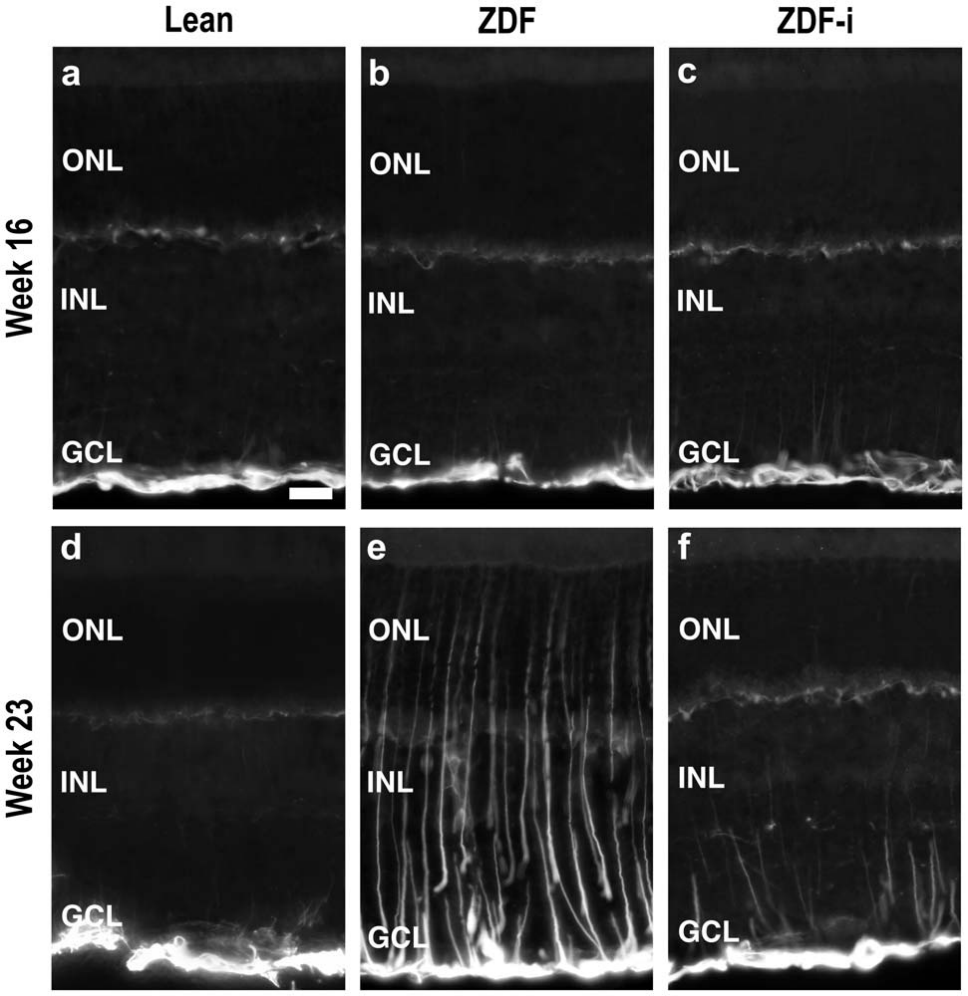
GFAP immunofluorescence. Localization of GFAP in cryostat sections obtained from Lean (**a**), ZDF (**b**) and insulin treated ZDF (**c**) rats at 16 weeks of age; and from Lean (**d**), ZDF (**e**) and insulin treated ZDF (**f**) rats at 23 weeks of age. ZDF-i, insulin-treated ZDFs; ONL, outer nuclear layer; INL, inner nuclear layer; GCL, ganglion cell layer. Scale bar = 20 µm.

In order to assess glial cell activation, retinal sections were processed for GFAP (glial fibrillary acidic protein). In Lean rats (Figs. 8a, 8d), GFAP expression was observed at all ages in the proximal retina, corresponding to the location of astrocytes and Müller cell endfeet, and in the far periphery also in the radial processes of Müller cells. In untreated ZDF rats, the same pattern of distribution was observed at 16 weeks of age (Fig. 8b), whereas at weeks 23 (Fig. 8e), 31 and 43, GFAP-labeled Müller cells were observed throughout the retina. In treated ZDF rats, no changes in GFAP distribution were observed 2 hours after the first injection (Fig. 8c), but after 7 weeks of daily insulin administration, labeling was less intense compared to untreated ZDFs (Fig. 8f).

## Discussion

In patients with diabetes, subnormal, supernormal as well as normal ERG responses have been observed [3], [16]–[20]. However, due to incomplete documentation of current and past glycemia a comparison of these studies is difficult [6], [8], [21]. In this study, an analysis of retinal function was performed in a naturally occurring type 2 diabetes model starting shortly after the onset of hyperglycemia. The evolution of ERG characteristics revealed features not previously reported in a diabetic animal. Photoreceptoral responses were of higher amplitude in the untreated diabetic animals as compared to congenic controls some weeks after the onset of hyperglycemia and fell abruptly within two hours of insulin treatment. Also noteworthy, inner retinal responses gradually decreased with age in untreated animals, despite sustained high photoreceptoral activity.

### Outer Retina

#### Hyperglycemic state

An age-related decrease in a-wave amplitudes is a common finding in rodents and has been reported to occur in different strains of rats at various ranges of ages beginning at 4 to 13 weeks of age and ending at 14 to 20 weeks of age [22]–[24]. Although not statistically significant, a reduction was similarly observed here in Lean rats after 8 weeks of age. ZDF rats, which develop fed hyperglycemia at about 6 weeks of age and, as shown here, fasted hyperglycemia after 10 weeks of age, did not however show such a reduction of the a-wave and exhibited amplitudes that were approximately 20% higher than those of Lean controls from 12 weeks. Measurement of the a-wave can be influenced by inner retinal activity since the photoreceptoral response is not complete before inner retinal responses begin, an issue which is particularly pertinent at low stimulus intensities. We found delays of the ZDF b-wave, but the differences in a-wave amplitudes as compared to Leans were most significant at the higher intensities where the a-wave implicit time was under 14 ms and therefore complete before the intrusion of any positive deflection from the inner retina [25], [26]. It appears then that the difference measured in a-wave amplitudes between Leans and ZDFs reflects a change in photoreceptoral function.

Most previous ERG studies have found a-wave amplitudes in diabetic rats to be similar or lower than controls [22], [27]–[31]. These seemingly conflicting results could be due to differences in the choice of strain, mode of diabetes induction, duration of diabetes, period of observation, and whether or not low-dose insulin was used to prevent wasting. A direct comparison with these studies is further complicated by the fact that the majority of them have been conducted on chemically induced type 1 models and have often used albino rat strains [32]–[34], which have a potentially confounding hereditary eye disease [29], [35]. Neuronal cell death has been observed in some such models [36], [37]. One study reported widespread TUNEL labeling in all layers of retinas from 26 week old the ZDF rats [38] and yet no reduction in retinal thickness, which should have occurred following extensive cell death, can be seen in the images provided. We found no signs of pathologic cell death by TUNEL labeling, at least during the study period (16–43 weeks of age), and also did not see significant thinning of the ZDF retinal layers as compared to Leans. It appears thus that cell loss is not responsible for the ERG alterations found here.

It is not clear what alterations are responsible for the higher amplitudes observed in ZDFs as compared to Leans. Larger responses occur in animals and humans after lead exposure as a result of increased number of rod photoreceptors and bipolar cells [39]. This is, however, observed only if lead exposure occurs prenatally, during a critical developmental period [39]–[40]. Hyperglycemia should not have such a neurogenic effect in ZDF rats since it ensues long after this period. Increased numbers of rods and/or bipolar cells could result also from alterations in the process of developmental cell death, but this is also completed before the onset of hyperglycemia [41]. Another possibility is if hyperglycemia were to cause perturbations in intracellular levels of Ca^2+^ and cGMP, which could increase the ZDF photoreceptoral amplitudes and sensitivity [42], [43]. Photoreceptors have a high energetic demand and it is conceivable that under chronic hyperglycemia, the excess available glucose [4], [5] is used to support or drive their responses. Over time, the ZDF photoreceptors appear to become dependent on this higher level of substrate, which was demonstrated here when glycemia was lowered.

#### Controlled glycemia

Unlike the increase in a-wave amplitudes, which took weeks to develop, the response to a sudden reduction of glucose levels was immediate. When insulin was first administered, the ZDF rats had been hyperglycemic for about 10 weeks. Two hours after the first injection, a- and b-wave amplitudes were prominently reduced when blood glucose was lowered to about 10 mmol/L.

Administration of exogenous insulin has been shown to increase retinal insulin receptor phosphorylation, and although insulin signaling in the retina is relatively insensitive to smaller fluctuations of circulating insulin [15], it could have had an effect on the ERGs. Insulin has been shown *in vitro* to suppress ERG responses independently of its effect on glycemia [44]. However, it has not been found to have a significant inhibitory effect *in vivo* [45], [46], which was confirmed in the present study, where the ERGs of Lean rats were found to be unaffected by insulin (Lean-i group). Also, at week 8, ZDF rats are hyperinsulinimic [11], and yet their responses at this time-point were not lower than those of Leans. While it is possible that ZDF and Lean rat retinas respond differently to systemic insulin, no difference was seen in immunofluorescence for phosphorylated Akt, a known downstream target of insulin signaling in the rat retina [15]. The large amplitude decreases noted here in the treated ZDF rats were therefore more likely caused by the reduction in glycemia than by a direct effect of insulin.

In treated ZDFs, glucose levels were still relatively high following the first insulin injection, but apparently not compatible with sustained, higher retinal activity. This may reflect how retinal function became dependent on high glucose levels during chronic hyperglycemia. Clinical electrophysiological and psychophysical studies of retinal function in patients with diabetes [6], [7], [47] suggest that relative hypoglycemia (normoglycemia after prolonged hyperglycemia) may be comparable to hypoglycemia in a healthy subject [48]. In the normal retina, small reductions in glycemia have little effect on photoreceptoral activity, which is maintained by a balance between both glycolysis and oxidative phosphorylation [49], [50]. In contrast, a diabetic retina appears not to utilize aerobic respiration to the same extent as a normal retina [51], [52], [48], perhaps due to a reduction in mitochondrial activity or number [53]. The prompt reduction of ZDF a-wave amplitudes in response to lowered glucose levels could reflect a similar shift in energy metabolism which rendered the retina abnormally dependent on glycolysis [51], [52]. Moreover, the amplitudes of the a-waves were not further affected by prolonged insulin treatment and the maximum a-wave slope was even lower compared to Lean rats at weeks 19 and 22. The persistently low responses indicate that the photoreceptors were unable to adapt to the new level of glycemia, at least after 6 weeks of treatment.

The observations made in the present study also underscore a difference between acute and chronic hyperglycemia. In this study, Lean rats showed no difference in a-wave amplitudes irrespective of whether glucose levels were high or normal (Lean vs. Lean-i). In the longitudinal study, however, although Lean and ZDF rats were both hyperglycemic while ERGs were recorded, it was not until after 4–6 weeks of sustained hyperglycemia that the differences in photoreceptoral responses became evident in ZDFs. These results indicate that acute hyperglycemia does not have an effect on photoreceptor responses in the normal retina, confirming previous studies in non-diabetic humans [54], [55], cats [56] and explants of normal rat retina [49].

### Inner Retina

The oscillatory potentials (OPs), understood to be governed by amacrine cell activity [57], revealed significantly prolonged ZDF implicit times after approximately 6–8 weeks of hyperglycemia. This feature has often been described in clinical reports [3], [58] and in rat models of type 1 diabetes [23], [24], [27], [28]. We also observed a delay of ZDF photopic b-waves, which could be the result of slower bipolar responses, or may simply be another expression of the delayed OPs, since these influence the shape of the photopic b-wave.

The magnitude of the scotopic b-wave is not only dependent upon the integrity of the inner retina but is also a function of the amplitude of the a-wave. A calculation of b/a ratios showed that the increase in ZDF b-wave amplitudes correlated well with the increase in photoreceptoral responses, at least initially. From 14 weeks of age, however, the ZDF b/a wave amplitude ratio declined continuously. Pathological changes in the inner retina of patients and other models of diabetes have been extensively documented and the gradual decrease in b-wave responsiveness found here is in accordance with such findings [23], [24], [28], [30], [31]. Retinal vascular perturbations have also been noted in ZDF rats [59]–[61] and there is therefore a possibility that such changes could contribute to the inner retinal changes detected here.

Up-regulation of GFAP in Müller cells has been demonstrated in the retinas of several models of diabetes and of diabetic patients and, as in other forms of pathology, is normally viewed as an indicator of retinal cell stress [62]–[64]. In the present study, GFAP levels were still normal at 16 weeks, while ERG abnormalities could be detected already from 10–12 weeks of age. The neuronal alterations that underlie the higher responses in ZDFs were therefore, at least initially, not sufficient to trigger increased GFAP expression. Over time, however, these alterations may have contributed to exhaust the homeostatic capacity of Müller cells. This, in combination with the sustained hyperglycemia, might have further impaired the function of these cells and their ability to support normal retinal physiology [1]. We did find widespread increased GFAP expression in Müller cells at 23 weeks in the retinas of ZDFs, suggestive of persistent stress on these and other cells in the retina. This notion is also supported by the fact that insulin administration at 16 weeks abated the up-regulation of GFAP. Insulin treatment resulted also in some recovery of the b/a ratio and in a stabilization of scotopic OP and photopic b-wave implicit times, which in untreated ZDFs became progressively slower.

In summary, the present study shows it is possible also in ZDFs to prevent, to a certain degree, a worsening of inner retinal function through glycemic control [65]. In addition, it shows that while prolonged hyperglycemia may have allowed photoreceptors in ZDFs to maintain a higher level of activity, it may also have rendered them dependent on high glycemic levels, a condition that was first perceived when blood glucose was lowered. We can only speculate about the reasons for this, but it is possible that in ZDFs, hyperglycemia altered the relative contributions of oxidative phosphorylation and glycolysis to overall energy production such that when glycemia was reduced, the photoreceptor cells experienced an energy supply crisis. In our studies of humans, we found that the retina eventually re-adapts to normoglycemia after periods of hyperglycemia, but that this is a protracted process [8]. If adaptation occurs in ZDFs, it may also be slow since we did not see any signs of recovery 6 weeks after starting insulin treatment.

The development of diabetes in the ZDF model occurs naturally and has much in common with type 2 diabetes in humans. Furthermore, the functional analysis performed here was started shortly after the onset of hyperglycemia, and therefore the findings of the present study are likely to reflect events that take place in pre-retinopathic stages but are often missed in clinical studies since a complete glycemic history is difficult to obtain. A clinical report by Tyrberg et al. [20] underscores this point by examining type 2 diabetics who had developed hyperglycemia at most 34 months prior to the study. This analysis of newly onset diabetes revealed a clear tendency towards higher full field ERG amplitudes in the dark-adapted state, resembling the results of the present study. It is therefore possible to characterize early aspects of retinal adaptation to hyperglycemia using electroretinography. In patients with diabetes, this may enable identification of the small subgroup of patients who will develop sight-threatening progression of diabetic retinopathy after initiation of improved metabolic control [9] and means of titrating therapy so that the problem can be reduced or eliminated.

## Supporting Information

Figure S1 **Group-averaged scotopic ERG traces.** Segments shown for stimuli of 0 log cd*s/m^2^ at weeks 16 (**a**, **b**) and 22 (**c**, **d**) depict differences between the a- and b-waves of ZDF and Lean rats (**a**, **c**) as well as ZDF and insulin-treated (ZDF-i) rats (**b**, **d**) in detail. The group-averaged oscillatory potentials for stimuli of 0 log cd*s/m^2^ at weeks 16 (**e**, **f**) and 22 (**g**, **h**) are overlaid to emphasize implicit time differences between ZDF and Lean rats (**e**, **g**) as well as ZDF and ZDF-i rats (**f**, **h**). ZDF-i, insulin-treated ZDFs.
(TIF)

Table S1 **Scotopic a-wave data.** Units for intensity denoted as log cd*s/m^2^; Data presented are group mean ±SD (see Table 1 for number of animals in each group at various ages); (A) Implicit times denoted in ms; (B) Amplitude denoted in µV; (C) Slope denoted in µV/0.4 ms; (A–C) Age denoted in weeks.
(PDF)

Table S2 **Scotopic b-wave amplitude data.** Units for intensity denoted as log cd*s/m^2^; Data presented are group mean ±SD (see Table 1 for number of animals in each group at various ages); Amplitude denoted in µV; Age denoted in weeks.
(PDF)

Table S3 **Scotopic b-wave implicit time data.** Units for intensity denoted as log cd*s/m^2^; Data presented are group mean ±SD (see Table 1 for number of animals in each group at various ages); Implicit times denoted in ms; Age denoted in weeks.
(PDF)

Table S4 **Scotopic oscillatory potential amplitudes.** Responses elicited by intensity of 0.0 log cd*s/m^2^; Data presented are group mean ±SD (see Table 1 for number of animals in each group at various ages); Age denoted in weeks.
(PDF)

Table S5 **Photopic b-wave data.** Units for intensity denoted as log cd*s/m^2^; Data presented are group mean ±SD (see Table 1 for number of animals in each group at various ages); Implicit times (A) denoted in ms; Amplitude (B) denoted in µV; Age denoted in weeks.
(PDF)
