# Supplementary figures and images for: Retinal Adaptation to Changing Glycemic Levels in a Rat Model of Type 2 Diabetes
Source: PLoS One. 2013 Feb 8;8(2):e55456. doi: 10.1371/journal.pone.0055456 (PMC3568153; doi:10.1371/journal.pone.0055456)

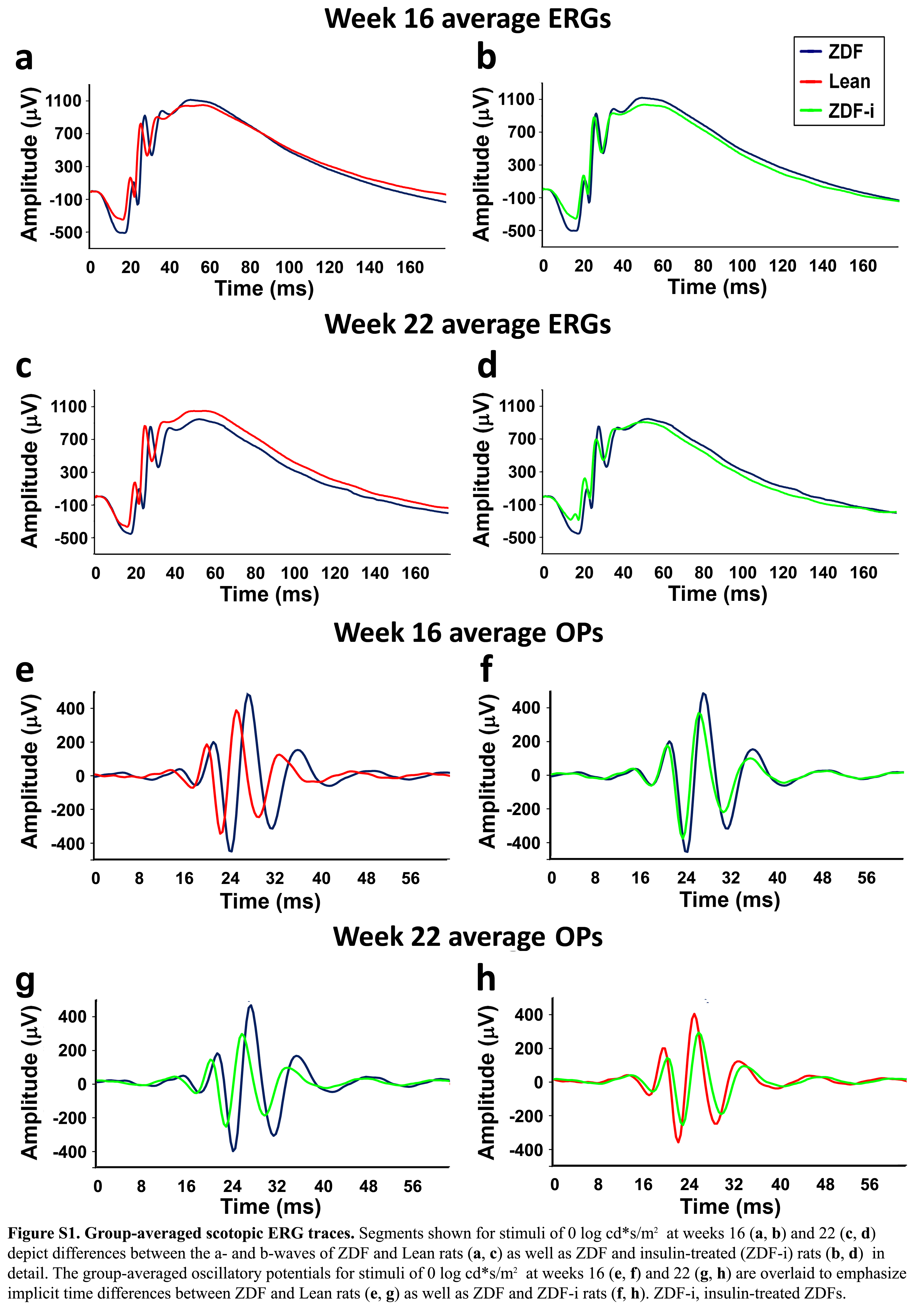

Supplement: Figure S1 — Group-averaged scotopic ERG traces. Segments shown for stimuli of 0 log cd*s/m2 at weeks 16 (a, b) and 22 (c, d) depict differences between the a- and b-waves of ZDF and Lean rats (a, c) as well as ZDF and insulin-treated (ZDF-i) rats (b, d) in detail. The group-averaged oscillatory potentials for stimuli of 0 log cd*s/m2 at weeks 16 (e, f) and 22 (g, h) are overlaid to emphasize implicit time differences between ZDF and Lean rats (e, g) as well as ZDF and ZDF-i rats (f, h). ZDF-i, insulin-treated ZDFs. (TIF) [file pone.0055456.s001.tif]
