# Supplementary material for: Retinal Adaptation to Changing Glycemic Levels in a Rat Model of Type 2 Diabetes
Source: PLoS One. 2013 Feb 8;8(2):e55456. doi: 10.1371/journal.pone.0055456 (PMC3568153; doi:10.1371/journal.pone.0055456)
Supplement: Table S1 — Scotopic a-wave data. Units for intensity denoted as log cd*s/m2; Data presented are group mean ±SD (see Table 1 for number of animals in each group at various ages); (A) Implicit times denoted in ms; (B) Amplitude denoted in µV; (C) Slope denoted in µV/0.4 ms; (A–C) Age denoted in weeks. (PDF) [file pone.0055456.s002.pdf]

Table S1. Scotopic a-wave data.

(A) Scotopic a-wave implicit times

**Intensity**	-1.0			0.0			0.5			1.0		
**Group**:	Lean	ZDF	ZDF-i	Lean	ZDF	ZDF-i	Lean	ZDF	ZDF-i	Lean	ZDF	ZDF-i
**Age**												
8	19.7±0.6	20±0.4		17.1±0.4	16.7±1.0		11.6±0.7	11.6±0.4		10.4±0.5	10.4±0.3	
10	19.1±0.2	**19.7±0.4 ***		16.9±0.3	17.1±0.5		11.4±0.2	11.7±0.4		10.3±0.3	10.5±0.3	
12	19.7±0.6	**20.1±0.5 ***		17.1±0.4	17.3±0.6		11.5±0.4	11.9±0.4		10.4±0.3	10.7±0.4	
14	18.7±0.6	**19.9±0.5** *		16.5±0.6	16.9±0.7		11.8±1.9	11.7±0.4		10.4±0.9	10.4±0.3	
16	18.5±0.6	**20.1±0.3** *	**19.6±0.4** †#	16.3±0.5	16.2±1.2	**17.1±0.4** †#	11.1±0.7	11.4±0.3	11.7±0.4	10.1±0.6	10.2±0.3	10.7±0.6
19	18.5±0.4	**20.2±0.4** *	**19.5±0.6** †#	16.2±0.4	**17.2±0.8** *	**16.9±0.5** †	10.9±0.5	**11.7±0.3** *	**12.4±1.7** †	9.8±0.4	10.5±0.4	10.5±0.4
22	18.7±0.2	**21.0±0.3** *	**19.9±0.6** †#	16.5±0.3	**17.9±0.8** *	**17.4±0.6** †	11.1±0.4	**12.2±0.3** *	14.0±1.9	10.2±0.3	10.8±0.3	11.2±0.3

(B) Scotopic a-wave amplitudes

**Intensity**	-1.0			0.0			0.5			1.0		
**Group**:	Lean	ZDF	ZDF-i	Lean	ZDF	ZDF-i	Lean	ZDF	ZDF-i	Lean	ZDF	ZDF-i
**Age**												
8	135±24	120±33		428±42	426±67		523±57	525±80		595±57	592±72	
10	106±31	123±25		389±62	423±56		459±69	520±62		523±86	582±74	
12	107±42	125±35		365±55	**452±75** *		425±96	**558±87** *		528±54	**628±90** *	
14	108±48	136±32		372±105	451±68		440±113	**551±66** *		497±109	**626±69** *	
16	106±29	**166±28** *	**98±30** #	348±53	**494±62** *	**360±61** #	413±70	**618±69** *	**411±74** #	468±78	**690±82** *	**472±92** #
19	121±30	128±26	**96±26** #	395±46	**449±48** *	**381±64** #	461±41	**544±56** *	**420±55** #	517±52	**616±56** *	**480±58** #
22	108±20	129±23	**82±22** #	372±38	**474±47** *	**324±59** #	434±37	**558±58** *	**362±78** #	487±36	**627±66** *	**422±86** #

(C) Maximal scotopic a-wave slope

**Intensity**	-1.0			0.0			0.5			1.0		
**Group**:	Lean	ZDF	ZDF-i	Lean	ZDF	ZDF-i	Lean	ZDF	ZDF-i	Lean	ZDF	ZDF-i
**Age**												
8	-8.9±2.3	-7.6±2.3		-22.7±4.1	-22.8±4.4		-43.0±7.4	-43.4±8.0		-61.8±8.6	-62.4±10.0	
10	-8.3±1.8	-7.8±1.8		-19.8±3.2	-22.1±3.1		-37.9±6.5	-41.9±5.7		-54.0±9.1	-60.1±7.1	
12	-7.4±2.1	-7.7±1.7		-19.1±4.3	**-23.4±5.3** *		-37.3±8.4	**-44.8±8.9** *		-55.9±9.0	**-64.3±11.4** *	
14	-8.5±3.2	-7.9±2.1		-20.3±6.1	-22.5±4.0		-38.1±10.9	-43.1±6.3		-53.5±14.0	**-62.6±8.9** *	
16	-9.0±4.5	-9.8±1.8	-6.9±2.0	-18.8±3.8	**-28.1±3.0** *	**-17.4±4.5** #	-35.3±7.1	**-52.3±6.1** *	**-33.6±8.7** #	-49.8±9.4	**-73.5±8.9** *	**-48.3±11.6** #
19	-9.2±2.8	-7.2±1.3	-6.7±2.0	-21.6±4.0	-22.6±3.2	**-16.4±4.4** †#	-41.0±6.6	-43.4±5.7	**-32.5±7.5** †#	-57.1±7.9	-62.7±7.1	**-48.5±10.4** †#
22	-8.4±2.0	-7.5±1.6	-6.9±1.3	-19.6±2.4	-22.4±3.2	**-14.2±3.7** †#	-37.4±4.8	-42.5±5.1	**-28.0±6.6** †#	-54.2±9.5	**-62.9±6.1** *	**-40.3±10.0** †#

Units for intensity denoted as log cd*s/m²; Data presented are group mean ±SD (see Table 1 for number of animals in each group at various ages); Implicit times (A) denoted in ms; Amplitude (B)denoted in μV; Slope (C) denoted in μV/0.4 ms; Age denoted in weeks.

ZDF, Zucker Diabetic Fatty rats; Lean, congenic control rats; ZDF-i, insulin treated ZDF

* p<0.05 between Lean and ZDF

† p<0.05 between Lean and ZDF-i

# p<0.05 between ZDF and ZDF-i
