# Supplementary material for: Retinal Adaptation to Changing Glycemic Levels in a Rat Model of Type 2 Diabetes
Source: PLoS One. 2013 Feb 8;8(2):e55456. doi: 10.1371/journal.pone.0055456 (PMC3568153; doi:10.1371/journal.pone.0055456)
Supplement: Table S2 — Scotopic b-wave amplitude data. Units for intensity denoted as log cd*s/m2; Data presented are group mean ±SD (see Table 1 for number of animals in each group at various ages); Amplitude denoted in µV; Age denoted in weeks. (PDF) [file pone.0055456.s003.pdf]

Table S2. Scotopic b-wave amplitude data.

(A) Scotopic b-wave amplitudes

Intensity	-3.7			-3.0			-2.0			-1.0		
**Group**:	Lean	ZDF	ZDF-i	Lean	ZDF	ZDF-i	Lean	ZDF	ZDF-i	Lean	ZDF	ZDF-i
**Age**												
8	496±99	442±117		894±191	881±155		1249±210	1288±164		1274±121	1284±153	
10	405±127	427±116		812±186	884±154		1200±206	1296±112		1188±203	1266±92	
12	391±100	430±108		789±142	898±146		1177±132	**1314±125** *		1153±117	**1263±117** *	
14	393±138	464±118		798±235	931±145		1136±235	**1293±131** *		1121±200	**1242±119** *	
16	366±94	495±143	401±63	761±130	986±151	839±123	1143±166	**1361±115** *	**1195±155** #	1143±152	**1319±123** *	**1164±143** #
19	399±117	434±51	348±104	804±123	890±49	748±160	1173±95	1275±85	**1104±129** #	1156±89	1217±68	**1067±92** †#
22	379±109	387±92	254±85	696±86	798±98	616±134	1102±103	1226±88	**989±164** †#	1091±99	1131±91	**1003±52** †#

	0.0			0.5			1.0		
	Lean	ZDF	ZDF-i	Lean	ZDF	ZDF-i	Lean	ZDF	ZDF-i
8	1587±112	1589±161		1628±125	1636±174		1610±117	1618±157	
10	1461±243	1566±114		1492±257	1610±145		1453±256	**1605±109** *	
12	1410±118	**1580±146** *		1489±133	**1649±153** *		1469±144	**1640±146** *	
14	1408±237	**1537±138** *		1436±208	**1576±134** *		1418±178	**1569±113** *	
16	1400±191	**1651±147** *	**1392±179** #	1449±203	**1714±153** *	**1402±183** #	1426±187	**1667±164** *	**1357±183** #
19	1421±83	1534±95	**1329±130** †#	1529±124	1590±100	**1365±145** †#	1413±68	1554±101	**1302±102** †#
22	1355±71	1458±102	**1265±85** †#	1453±79	1487±113	**1273±90** †#	1379±71	1481±122	**1227±78** †#

(B) Scotopic b/a amplitude ratio

Intensity	-1.0			0.0			0.5			1.0		
**Group**:	Lean	ZDF	ZDF-i	Lean	ZDF	ZDF-i	Lean	ZDF	ZDF-i	Lean	ZDF	ZDF-i
**Age**												
8	9.6±1.2	11.4±2.8		3.7±0.1	3.8±0.3		3.1±0.1	3.1±0.3		2.7±0.1	2.7±0.2	
10	12.0±3.3	10.6±1.9		3.8±0.3	3.7±0.3		3.2±0.2	3.1±0.2		2.8±0.1	2.8±0.3	
12	12.5±5.9	10.5±1.8		3.9±0.4	**3.5±0.3** *		3.2±0.2	3.0±0.2		2.8±0.2	2.6±0.2	
14	10.0±1.6	9.9±1.7		3.7±0.2	**3.5±0.2** *		3.2±0.2	**2.9±0.2** *		2.8±0.1	**2.5±0.2** *	
16	11.4±3.0	8.1±1.3	12.8±3.7	4.0±0.2	**3.4±0.3** *	**3.9±0.3** #	3.5±0.3	**2.8±0.1** *	**3.4±0.4** #	3.1±0.2	**2.4±0.2** *	**2.9±0.3** #
19	10.4±2.3	9.8±1.7	11.8±2.9	3.8±0.2	**3.4±0.2** *	3.5±0.3	3.3±0.1	**2.9±0.2** *	**3.3±0.2** #	2.9±0.2	**2.5±0.2** *	**2.7±0.2** †#
22	10.9±1.6	9.0±1.4	13.1±4.2	3.8±0.3	**3.1±0.2** *	**4.0±0.5** #	3.4±0.2	**2.7±0.2** *	**3.6±0.6** #	2.9±0.2	**2.4±0.2** *	**3.0±0.5** #

Units for intensity denoted as log cd*s/m²; Data presented are group mean ±SD (see Table 1 for number of animals in each group at various ages); Amplitude denoted in μV; Age denoted in weeks.

ZDF, Zucker Diabetic Fatty rats; Lean, congenic control rats; ZDF-i, insulin treated ZDF

* $p<0.05$ between Lean and ZDF

† $p<0.05$ between Lean and ZDF-i

# $p<0.05$ between ZDF and ZDF-i
