# Supplementary material for: Retinal Adaptation to Changing Glycemic Levels in a Rat Model of Type 2 Diabetes
Source: PLoS One. 2013 Feb 8;8(2):e55456. doi: 10.1371/journal.pone.0055456 (PMC3568153; doi:10.1371/journal.pone.0055456)
Supplement: Table S3 — Scotopic b-wave implicit time data. Units for intensity denoted as log cd*s/m2; Data presented are group mean ±SD (see Table 1 for number of animals in each group at various ages); Implicit times denoted in ms; Age denoted in weeks. (PDF) [file pone.0055456.s004.pdf]

Table S3. Scotopic b-wave implicit time data.

Scotopic b-wave implicit times

**Intensity**	-3.7			-3.0			-2.0			-1.0		
**Group**:	Lean	ZDF	ZDF-i	Lean	ZDF	ZDF-i	Lean	ZDF	ZDF-i	Lean	ZDF	ZDF-i
**Age**												
8	88.7±3.5	89.4±3.9		84.7±2.5	85.1±3.0		68.6±1.9	70.1±3.3		50.7±1.1	51.3±1.3	
10	88.3±3.0	88.7±3.1		82.2±2.9	84.5±2.8		69.5±2.3	70.1±2.9		50.9±1.1	50.9±1.5	
12	88.4±4.5	**92.9±3.9** *		85.9±2.3	86.2±3.0		70.7±3.7	71.4±2.9		51.6±1.9	51.4±1.2	
14	85.5±2.9	**91.3±2.8** *		83.5±2.8	**86.4±3.2** *		67.9±5.4	69.0±2.7		51.7±3.6	51.1±1.7	
16	86.6±5.3	**94.4±4.4** *	**89.5±1.6** #	82.8±3.9	**87.2±5.0** *	**87.0±1.4** †	67.7±4.4	68.7±5.2	69.9±2.6	48.3±1.3	**51.6±1.7** *	**51.3±1.8** †
19	83.7±3.1	**92.2±4.3** *	**89.0±4.4** †	83.0±2.8	86.0±3.7	**86.3±4.3** †	66.4±3.4	69.4±2.4	69.6±2.8	48.8±1.6	50.4±0.9	49.2±3.5
22	86.2±2.3	**93.9±2.7** *	**88.5±3.5** #	82.3±2.7	**89.9±3.9** *	**82.1±7.2** #	67.8±2.0	72.4±3.0	68.7±2.9	49.4±1.7	**52.7±2.0** *	**50.6±1.1** #

	0.0			0.5			1.0		
	Lean	ZDF	ZDF-i	Lean	ZDF	ZDF-i	Lean	ZDF	ZDF-i
8	51.4±2.1	51.6±2.1		50.6±1.9	50.8±1.5		51.0±1.8	51.4±1.4	
10	53.1±2.6	**50.9±1.5** *		51.3±2.4	51.1±1.9		51.5±2.4	51.5±1.5	
12	52.8±1.4	50.9±1.5		51.8±1.4	50.7±1.8		52.9±1.7	51.5±1.6	
14	54.0±2.1	**50.8±1.7** *		51.8±2.2	50.9±1.7		51.2±2.0	51.9±1.6	
16	50.0±4.3	50.9±2.7	52.0±1.9	49.8±5.0	50.7±3.0	51.5±1.3	48.6±3.6	**52.1±3.2** *	**52.6±1.2** †
19	48.0±1.6	**50.4±1.0** *	**52.2±2.5** †	47.5±1.7	**50.4±1.3** *	**52.8±2.9** †#	48.1±1.8	**51.7±1.5** *	**52.9±2.4** †
22	50.2±3.1	**52.8±2.2** *	**53.9±2.1** †	49.1±2.0	**53.1±2.4** *	**52.6±1.3** †	49.5±2.0	50.5±6.0	**53.2±1.6** †

Units for intensity denoted as log cd*s/m^2; Data presented are group mean ±SD (see Table 1 for number of animals in each group at various ages); Implicit times denoted in ms; Age denoted in weeks.

ZDF, Zucker Diabetic Fatty rats; Lean, congenic control rats; ZDF-i, insulin treated ZDF

* $p<0.05$ between Lean and ZDF

† $p<0.05$ between Lean and ZDF-i

# $p<0.05$ between ZDF and ZDF-i
