# Supplementary material for: Retinal Adaptation to Changing Glycemic Levels in a Rat Model of Type 2 Diabetes
Source: PLoS One. 2013 Feb 8;8(2):e55456. doi: 10.1371/journal.pone.0055456 (PMC3568153; doi:10.1371/journal.pone.0055456)
Supplement: Table S4 — Scotopic oscillatory potential amplitudes. Responses elicited by intensity of 0.0 log cd*s/m2; Data presented are group mean ±SD (see Table 1 for number of animals in each group at various ages); Age denoted in weeks. (PDF) [file pone.0055456.s005.pdf]

Table S4. Scotopic oscillatory potential amplitudes.

Scotopic OP amplitude/maximal a-wave slope

**OP number:**	1			2		
**Group**:	Lean	ZDF	ZDF-i	Lean	ZDF	ZDF-i
**Age**						
8	13.6±0.8	11.0±1.2		41.6±2.3	38.3±4.4	
10	13.5±1.2	10.8±1.1		39.2±2.1	37.7±3.0	
12	12.8±2.5	9.7±1.5		38.8±6.7	35.2±5.1	
14	13.9±2.1	9.9±1.4		38.5±5.9	37.1±5.1	
16	14.5±1.8	9.3±1.3	13.7±2.3	40.5±5.7	34.2±4.0	42.6±6.4
19	14.0±1.7	11.7±1.5	14.9±2.5	38.2±3.0	41.1±4.2	44.4±8.0
22	13.5±2.3	10.7±2.0	14.9±3.4	37.8±5.0	37.7±7.1	46.3±5.3

	3			4		
	Lean	ZDF	ZDF-i	Lean	ZDF	ZDF-i
8	18.3±2.8	18.4±3.0		3.8±0.4	3.5±0.4	
10	15.9±2.3	17.7±2.5		3.4±0.4	3.5±0.4	
12	16.5±4.3	17.4±2.6		3.4±0.8	3.3±0.5	
14	16.5±3.9	**19.5±3.2** *		3.5±0.6	3.8±0.7	
16	20.7±3.5	**17.7±4.0** *	19.8±3.3	3.8±0.6	3.5±1.1	4.2±0.7
19	18.7±2.5	21.5±3.0	20.4±4.9	3.8±0.5	4.1±0.6	3.9±0.7
22	17.4±3.2	18.8±3.5	**24.1±4.3** †#	3.8±0.5	3.8±0.5	4.3±1.1

Responses elicited by intensity of 0.0 log cd*s/m²; Data presented are group mean ±SD (see Table 1 for number of animals in each group at various ages); Age denoted in weeks.
OP, oscillatory potential; ZDF, Zucker Diabetic Fatty rats; Lean, congenic control rats; ZDF-i, insulin treated ZDF
* $p<0.05$ between Lean and ZDF
† $p<0.05$ between Lean and ZDF-i
# $p<0.05$ between ZDF and ZDF-i
