# Supplementary material for: Retinal Adaptation to Changing Glycemic Levels in a Rat Model of Type 2 Diabetes
Source: PLoS One. 2013 Feb 8;8(2):e55456. doi: 10.1371/journal.pone.0055456 (PMC3568153; doi:10.1371/journal.pone.0055456)
Supplement: Table S5 — Photopic b-wave data. Units for intensity denoted as log cd*s/m2; Data presented are group mean ±SD (see Table 1 for number of animals in each group at various ages); Implicit times (A) denoted in ms; Amplitude (B) denoted in µV; Age denoted in weeks. (PDF) [file pone.0055456.s006.pdf]

Table S5. Photopic b-wave data.

(A) Photopic b-wave implicit times

**Intensity:**	0.0			0.5			1.0		
**Group:**	Lean	ZDF	ZDF-i	Lean	ZDF	ZDF-i	Lean	ZDF	ZDF-i
**Age**									
8	30.2±0.9	30.8±.1		36.7±0.4	36.9±1.0		35.8±0.5	36.5±1.1	
10	31.8±4.4	30.3±1.0		36.3±0.4	37.1±0.6		35.8±0.4	36.7±0.9	
12	29.4±1.1	30.3±0.9		36.1±0.2	**37.1±0.5** *		35.2±0.5	**36.4±0.4** *	
14	30.6±4.1	30.0±1.0		35.7±0.7	**37.4±0.8** *		34.9±0.7	**36.3±1.3** *	
16	29.9±1.1	30.3±0.6	30.2±0.6	36.2±0.5	**38.5±0.9** *	**37.8±1.0** †	35.5±0.8	**37.6±0.7** *	**37.1±0.9** †
19	31.2±4.2	30.8±0.6	30.2±1.5	36.0±0.7	**38.4±0.6** *	**37.7±0.7** †	34.8±0.9	**37.9±0.7** *	**37.1±0.7** †
22	29.4±0.6	31.1±0.7	31.2±3.6	36.1±0.8	**39.9±0.9** *	**37.5±0.8** †#	35.1±0.9	**39.4±1.0** *	**37.6±1.3** †#

	1.3			1.5		
	Lean	ZDF	ZDF-i	Lean	ZDF	ZDF-i
8	46.7±0.9	47.1±1.8		44.5±1.3	44.6±1.7	
10	45.8±1.0	47.0±1.1		44.0±0.9	45.0±1.1	
12	45.5±1.3	47.1±0.8		43.0±0.2	**44.9±0.9** *	
14	44.9±1.3	**48.5±1.0** *		43.3±1.5	**46.1±1.2** *	
16	42.2±5.8	**49.9±1.7** *	**49.8±1.4** †	43.5±1.4	**47.2±1.2** *	**47.1±1.2** †
19	45.2±2.2	**49.6±1.7** *	**49.6±1.5** †	42.8±2.4	**47.6±0.8** *	**46.7±1.7** †
22	45.2±1.2	**52.3±1.8** *	**48.4±2.2** †#	43.1±1.7	**50.3±1.9** *	**45.5±1.4** †#

(B) Photopic b-wave amplitudes

**Intensity**	0.0			0.5			1.0		
**Group**:	Lean	ZDF	ZDF-i	Lean	ZDF	ZDF-i	Lean	ZDF	ZDF-i
**Age**									
8	84±12	89±12		224±34	224±42		291±21	289±46	
10	81±13	84±18		194±50	199±52		249±49	265±63	
12	83±9	82±14		217±20	224±38		260±40	298±38	
14	79±15	78±12		208±28	210±30		256±25	268±38	
16	73±18	89±7	80±19	189±35	214±31	197±33	232±35	284±33	229±27
19	84±11	85±11	75±12	224±33	213±40	188±38	268±36	266±32	212±38
22	80±11	80±10	65±9	214±18	203±31	175±16	248±21	241±31	218±24

	1.3			1.5		
	Lean	ZDF	ZDF-i	Lean	ZDF	ZDF-i
8	305±23	293±48		308±19	313±50	
10	241±52	**296±63** *		265±55	306±53	
12	284±32	310±36		283±20	334±41	
14	257±12	270±33		279±36	285±38	
16	252±45	**297±40** *	**242±40** #	268±27	302±37	251±34
19	291±31	281±39	**205±49** †#	299±31	279±39	222±42
22	275±24	250±44	239±18	283±21	278±61	254±8

Units for intensity denoted as log cd*s/m²; Data presented are group mean ±SD (see Table 1 for number of animals in each group at various ages); Implicit times (A) denoted in ms; Amplitude (B) denoted in μV; Age denoted in weeks.

ZDF, Zucker Diabetic Fatty rats; Lean, congenic control rats; ZDF-i, insulin treated ZDF

* p<0.05 between Lean and ZDF

† p<0.05 between Lean and ZDF-i

# p<0.05 between ZDF and ZDF-i
